# Down-regulation of Xist and Mir-7a-5p improves LPS-induced myocardial injury

**DOI:** 10.7150/ijms.45408

**Published:** 2020-09-16

**Authors:** Dongshi Liang, Yimei Jin, Miaomiao Lin, Xiaojiao Xia, Xiaoli Chen, Airong Huang

**Affiliations:** The Second Affiliated Hospital of Wenzhou Medical University, Wenzhou, China.

**Keywords:** Xist, Mir-7a-5p, Sepsis, Myocardial cells

## Abstract

**Background:** X-inactive specific transcript (Xist) is a lncRNA, which plays a significant role in X-chromosome inactivation, regulates cell proliferation in tumor cells, and inhibits apoptosis in acute myocardial infarction. On the other hand, miR-7a-5p is involved in cardiomyocytes injury in myocardial ischemia/reperfusion. However, their roles in LPS-induced damage remain unclear.

**Objectives:** This study aimed at using siRNA transfection and lentivirus infection to regulate the expression of xist and miR-7a-5p, and to evaluate their effects on LPS-induced myocardial damage.

**Method:** Mice cardiomyocytes (MCM) cells were divided into six groups, namely the control group, the LPS group, the LPS + lncRNA^-^ group, the LPS + lncRNA^+^ group, the LPS + miRNA^-^ group, and the LPS + miRNA^+^ group. Quantitative real-time PCR (qRT-PCR) was performed to assay for the RNA expressions of xist, miR-7a-5p, peroxisome proliferator-activated receptor-γ coactivator-1α (PGC-1α), and recombinant mitochondrial transcription factor A (Tfam) in all the groups. The ATP level was determined using the adenosine triphosphate (ATP) assay kit according to the manufacturer's instructions. Flow cytometry was performed to estimate the level of apoptosis and proliferation in cells in each group.

**Results:** The level of xist in the myocardial cells was markedly higher in the LPS group compared with the control group; however, it was reduced in the LPS+ lncRNA^-^ group. There was no significant difference in the expression of xist among the LPS+miRNA^-^, LPS+miRNA^+^, and LPS groups. Moreover, the expression of mir-7a-5p was significantly reduced in myocardial cells in the LPS group, and moderately reduced in the LPS+ miRNA^-^ group, but remarkably elevated in the LPS+ miRNA^+^ group (P<0.05). The expression of mir-7a-5p was comparably similar in the LPS+ lncRNA^-^ group, LPS+ lncRNA^+^ group, and LPS groups. Further, the levels of PGC-1a, and Tfam were determined. In the LPS group, the expression of PGC-1α was significantly reduced but elevated in the LPS+lncRNA^-^ and LPS+ miRNA^-^ groups (P<0.05). There was no significant difference in the level of PGC-1α among the LPS, LPS+ lncRNA^+^, and LPS+ miRNA^+^ groups. The expression of Tfam was markedly reduced in the LPS group (*P <* 0.05), but elevated after the suppression of xist and mir-7a-5p. The expression of Tfam was not significantly different among the LPS group, LPS+ lncRNA^+^ and LPS+ miRNA^+^ groups. Notably, overexpression of mir-7a-5p had a mild effect on the expression of Tfam in the LPS+ miRNA^+^ group compared with the control group. Besides, ATP expression in the LPS group was markedly reduced, but elevated after the inhibition of xist and mir-7a-5p. Suppressing the expression of xist or mir-7a-5p resulted in reduced cell apoptosis and increased cell proliferation.

**Conclusions:** In this study, we established that down-regulation of xist and mir-7a-5p reduces apoptosis in response to LPS.

## Introduction

Sepsis has been recognized as a leading cause of human diseases and death among critically ill patients 1. Severe-sepsis-associated mortality rates range from 34% to 56% 2. Myocardial dysfunction is common in severe sepsis, and microcirculatory and mitochondrial dysfunctions play an essential role in the development of severe sepsis 3,4. Currently, the pathogenesis of sepsis-induced myocardial damage remains unclear, and thus its treatment remains a challenge. Findings from previous studies indicate that various inflammatory and non-coding RNA are involved in sepsis-induced myocardial damage through modulation, and the latter has received increased research attention in recent years 6,7.

Long noncoding RNAs (lncRNAs) are a class of RNA that are longer than 200 nucleotides, and with no protein-coding potential 8. They modulate gene expression at different levels, including chromosome reconstruction, transcription, and post-transcription 9. LncRNAs play various critical biological functions such as acting as signaling, decoy, guide, and scaffold molecules 10. For instance, they participate in the modulation of the occurrence and development of various cardiovascular diseases 11. LncRNA NEAT1 relieves sepsis-induced myocardial injury by modulation the TLR2/NF-κB signaling cascade 12. In rat models with acute myocardial infarction, the knockdown of xist represses myocardial cell apoptosis by modulating miR-449 13. Xist is a 17-kb lncRNA that regulates X chromosome inactivation in mammals resulting in equivalence in gene dosage between the XX female and the XY male 14. In recent studies, xist has been shown to target corresponding miRNAs and regulate the pathological process of various diseases. Xist protects cardiomyocyte hypertrophy by targeting miR-330-3p and modulates myocardial infarction by targeting miR-130a-3p†^15^. However, the mechanism of xist in sepsis -induced myocardial damage remains unclear.

MicroRNAs (miRNAs) are a kind of RNA about 19-24 nucleotides long, with no protein-coding potential 16. They play essential roles in biological processes by acting as key modulators inhibitors of gene expression by binding the 3' untranslated region (UTR) to suppress translation 17,18. Recent studies have shown that miRNAs are involved in sepsis-induced myocardial depression 19. MiR-7a-5p miRNA is 22 nucleotides long in its mature form 20 and improves ischemic brain damage by repressing α-synuclein 21. The delta-opioid receptor (DOR) is activated in myocardial ischemia/reperfusion 22. Moreover, DOR activation further increases the level of miR-7a-5p in hypoxia-induced heart, which has protective effects on myocardial ischemia injury 23. Accumulating evidence shows that mir-7a-5p down regulates IL-5 activated by IL-1B in Parkinson's disease and reduces neuro-inflammation and apoptosis 24. However, the mechanism of miR-7a-5p in sepsis-induced myocardial damage is unknown. Therefore, in this study, the mechanism of xist and miR-7a-5p in sepsis-induced myocardial damage was determined. The results revealed that elevated expression of xist and miR-7a-5p are characteristic molecular variations in sepsis-induced myocardial damage. Moreover, the suppression of xist and miR-7a-5p was speculated to improve sepsis-induced myocardial damage. This study aimed at elucidating the regulation mechanisms of xist and miR-7a-5p in sepsis-induced myocardial damage to help advance the understanding of sepsis-induced myocardial damage and provide new insights for its clinical treatment.

## Materials and Methods

### Cell harvesting

Mouse cardiomyocytes MCM cells were obtained from Otwo Biotech (Shenzhen, China). The Mouse cardiomyocytes MCM cells were cultured in Dulbecco's modified eagle medium supplemented with 10% fetal bovine serum, 1% streptomycin, and penicillin and incubated at 37°C and 5% CO2. When the degree of cell fusion reached 70-80%, the old medium was we replaced with fresh complete culture medium containing 1 µg/ml lipopolysaccharides (LPS) 10,25 and incubated for another 24 hours. The cells were then divided into six groups as follows (3 samples in each group): (a) the control group, in which the cells received completed culture media without LPS. (b) The LPS group, LPS (1 µg/ml) added and incubated for 24 hours. (c) LPS+ lnc RNA^-^ group, the cells were incubated with LPS (1 µg/ml) for 24 hours, and siRNA interference performed to inhibit the expression of xist, and incubation for another 48 hours. (d) LPS + lnc RNA^+^ group, where the MCM cells were incubated with LPS (1 µg/ml) for 24 hours, lentivirus vector expressing xist added, followed by incubation for 48 hours. (e) LPS + miRNA^-^ group, where the MCM cells were incubated with LPS (1 µg/ml) for 24 hours, followed by siRNA interference to inhibit the expression of mir-7a-5p, and incubation of 48 hours. (f) LPS + miRNA^+^ group, where the MCM cells were incubated with LPS (1 µg/ml) for 24 hours, and the mir-7a-5p mimic introduced, followed by incubation for 48 hours. Cells were collected from cell culture plates at the same time point.

### 2. RNA interference

Small interfering RNA (siRNA) targeting xist was designed and constructed by Biomedicine biotech (Chongqing, China). The mir-7a-5p inhibitor was obtained from Biomedicine biotech (Chongqing, China). MCM cells overexpressing xist were constructed using lentiviral approaches. Lentiviral vectors expressing xist were purchased from Biomedicine biotech (Chongqing, China). The overexpressing mir-7a-5p mimic was used.

### Cell transfection

Transfection was performed using the Nanofusion version 2.0 Transfection kit (Biomedicine, China), according to the manufacturer's instructions. Xist and mir-7a-5p inhibitors or mir-7a-5p mimic were transected at a final concentration of 60 uM for 6 hours, and the old media replaced with complete media. Cell transfection lasted for 48 hours.

### Lentivirus infection

The old medium was replaced with fresh medium containing 6 µg/ml polybrene, then added the virus suspension, and incubated at 37ºC for 4 hours. About, 2 ml fresh medium was added to dilute polybrene and the cells used for further experiments, within 48 h after transfection. The LPS + lnc RNA ^+^ group was established using the lentivirus infection.

### Determination of cell apoptosis and proliferation

Cell apoptosis and proliferation were assessed using flow cytometry (FCM) (BD Biosciences, United States). The MCM cells were collected after transfection for 48 hours and washed twice using PBS. The cells were harvested using trypsin without EDTA and collected after centrifugation at 2000rpm for 3 minutes. Subsequently, the cells were washed using ice-cold PBS, the cells collected, and added into the apoptosis analysis solution. Apoptotic cells were labeled using Annexin V-FITC and propidium iodide (PI) (E-CK-A217, Elabscience) following the manufacturer's instructions. Similarly, flow cytometry was used to estimate cellular proliferation. The cells in different groups were incubated at a final concentration of 10 µM BrdU for 1 hour. The cells were harvested and re-suspended at a density of 2×10^7^ cells/ml. The cells were fixed and permeabilized, the medium removed, the medium and BrdU incorporated. The newly synthesized DNA was quantified by flow cytometry (grouped as follows: Control, LPS, LPS+ lncRNA^-^, LPS+ lncRNA^+^ / Control, LPS, LPS+ miRNA^-^, LPS+ miRNA^+^).

### ATP analysis

Sample preparation: The medium was removed, and a total of 200 µl of lysate was added to a six-well plate. After lysis, the cells were centrifuged at 12,000 rpm for 5 min at 4°C. To prepare a standard curve, the ATP standard solution was used. Specific concentrations used were 0, 0.1, 0.3, 1, 3 and 10 μM. (See Supplementary Materials1.). The working solution for ATP testing was prepared by taking an appropriate amount of ATP detection reagent and dilute with the ATP detection reagent diluent at a ratio of 1:9 and the procedure was carried out on the ice. To determine the ATP concentration, 100 μL of ATP detection working solution was added to the well and incubated at room temperature for 3-5 min, so that all the background ATP was consumed. About 20 μL of the sample or standard was added to the test well, quickly mixed using a gun, and at least 2 seconds apart, a luminometer was used to measure the RLU value.

### RNA extraction and quantitative real-time PCR

Total RNA was isolated from the cells using the TRIzol reagent according to the manufacturer's instructions. MRNA expression was evaluated by quantitative real‐time PCR using SYBR Green (ABI, USA). The mRNA expression levels of xist, mir-7a-5p, PGC-1α, and Tfam were quantified. The primer sequences of these genes are shown in **Table 1.** The PCR conditions were set as follows; 95ºC for 30s, 95ºC for 5s, and 60ºC for 30-34s, a total of 40 cycles. All the samples were analyzed in triplicates. The relative expression levels of the genes were calculated using the 2^-ΔΔCt^ method.

### Western blotting

Proteins were extracted from the protein lysate, and their concentrations determined using the BCA kit (Takara, Japan). The protein samples were mixed with the buffer in a 4:1 ratio, the protein concentration kept constant at 3.2 μg/μl, and Western blotting performed. Protein samples (64 μg) were separated using a 4-20% gradient SDS-PAGE gel. The samples were incubated overnight at 4ºC (rabbit anti-rat, PGC-1a, Tfam concentration were 1:1000-2000, 18-24 hours). The next day, the samples were incubated with the secondary antibody (1:1000) for 1 hour. The reaction time was 1 minute and the chemiluminescence detection reagent (reagent A: reagent B = 1:1) was used.

### Statistical analyses

Data were analyzed using the Statistical Product for Social Sciences (SPSS; version 20.0). The data were presented as mean ± standard deviation (SD). For the ATP level and q-PCR assays, the results were analyzed using a one-way analysis of variance. *P*-value <0.05 was considered statistically significant.

## Results

### Establishment of the sepsis cell model

The morphology of the myocardial cells cultured was evaluated under an ordinary optical microscope (100×). (See Supplementary Materials 2 for details).

### Transfection Efficiency

**Figure 1:**
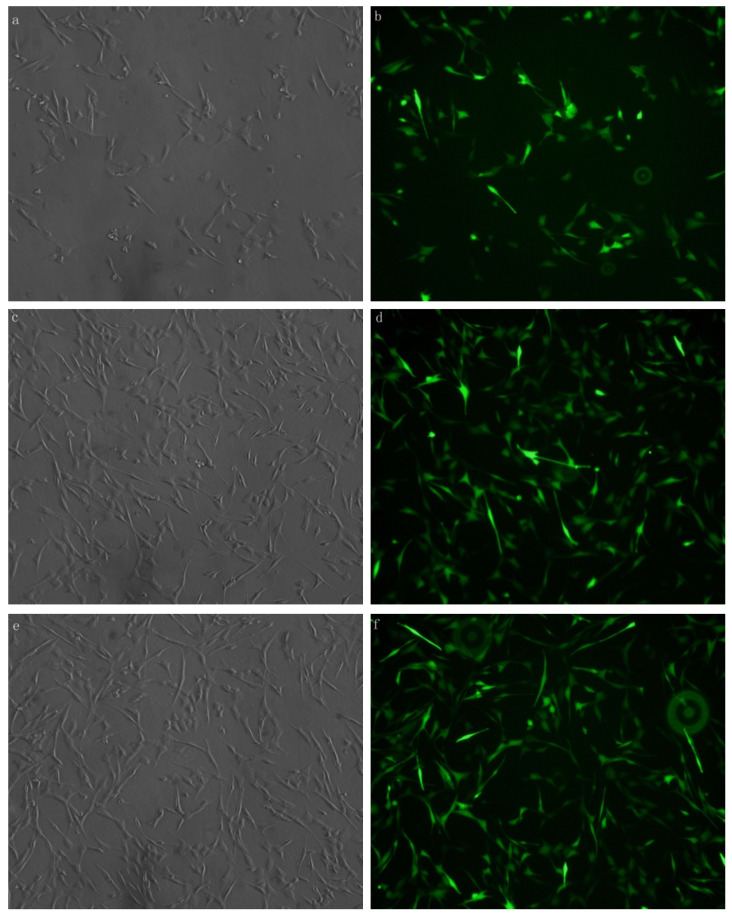
MCM cells were transfected with xist expression lentivirus or control vector lentivirus. Transfection was successful and reliable. a: Brightfield image of xist siRNA 100× b: Dark-field image of mXist siRNA 100×; c: Bright field image: overexpression of xist in LPS+ lncRNA^+^ group; d: Dark-field image: overexpression of xist in LPS+ lncRNA^+^ group; e: Bright field image: control vector lentivirus in LPS+ lncRNA^+^ group; f: Dark-field image : control vector lentivirus in LPS+ lncRNA^+^ group.

Xist was transfected with fluorescent markers into the myocardial cells aid in detecting the efficiency of transfection. Green fluorescence labeling was detected in the cells. The results showed that xist siRNA or xist over-expression vector was successfully transfected into myocardial cells (**Figure 1**).

### Effects of xist and mir-7a-5p on the myocardial cells

**Figure 2:**
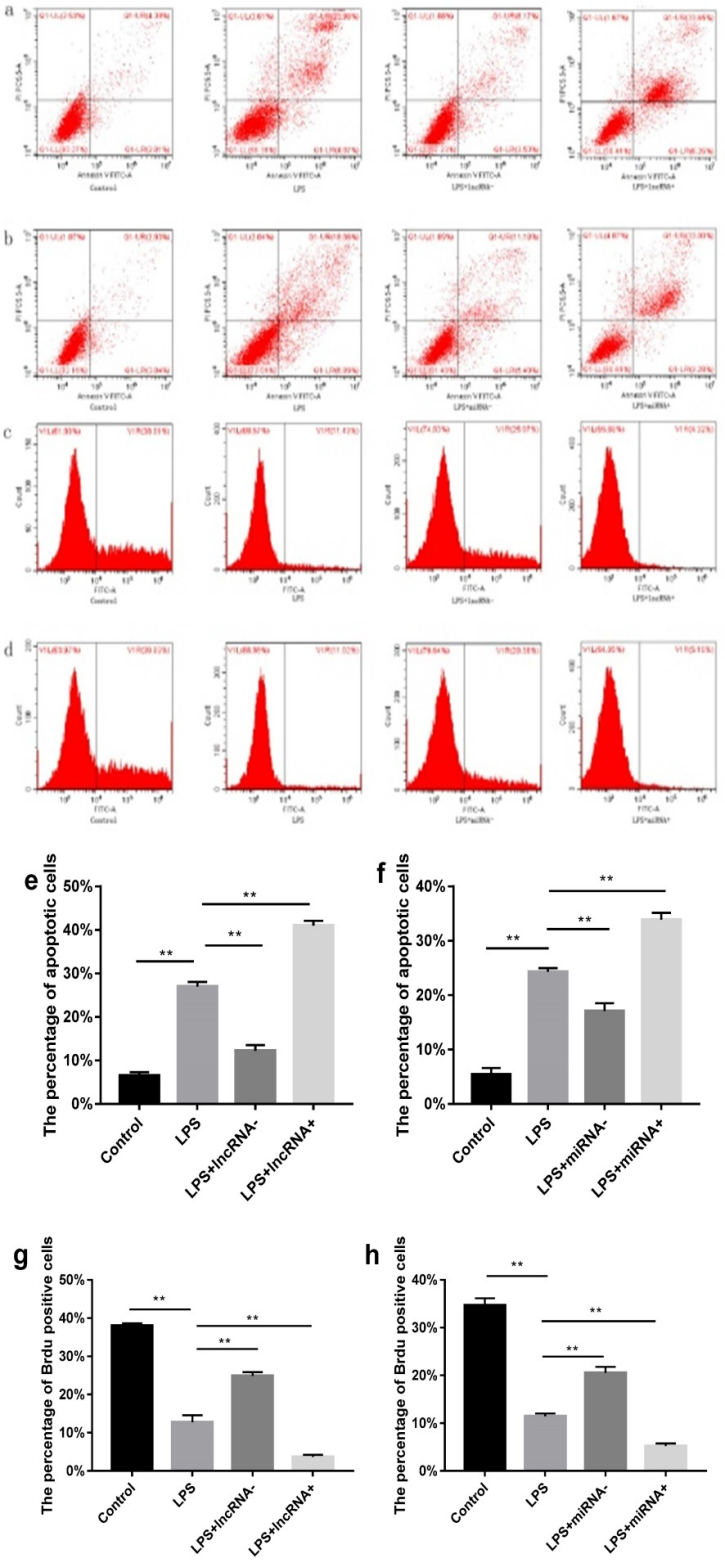
Effect of xist and mir-7a-5p on cell apoptosis and proliferation. a and e show the effect of xist on apoptosis in cells determined using flow cytometry. b and f illustrate the effects of mir-7a-5p on apoptosis in cells. Effect of xist/ mir-7a-5p on cell proliferation (c, g / d, h), **p<0.01.

Flow cytometry was performed to estimate the level of apoptosis and proliferation in cells in each group. Compared with the control group, a higher percentage of apoptotic cells and a low proliferation rate was found in the LPS group. Suppressing the expression of xist resulted in reduced cell apoptosis and increased cell proliferation. However, the opposite effect was observed following xist overexpression. Repressing the expression of mir-7a-5p, reduced cell apoptosis, and increased cell proliferation. In contrast, overexpression of mir-7a-5p induced opposite effects on cell apoptosis and proliferation (**Figure 2**).

### Suppression or overexpression of xist does not affect the expression of mir-7a-5p

**Figure 3:**
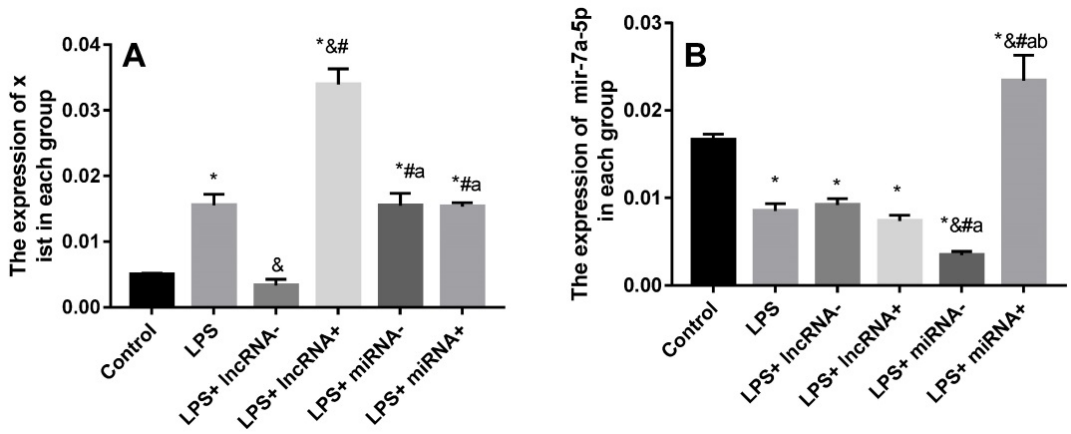
Expression levels of xist and mir-7a-5p in each group. A: The expression of xist is elevated after LPS treatment. Mir-7a-5p inhibition or mir-7a-5p overexpression had no impact on the expression of xist. B: The expression of mir-7a-5p is reduced after LPS treatment. Inhibition or overexpression of xist did not alter mir-7a-5p expression levels. *p < 0.05 compared with the control group; & compared with the LPS group; # compared with the LPS+ lncRNA^-^ group; a compared with the LPS+ lncRNA^+^ group; b compared with the LPS+ miRNA^-^ group.

SiRNA or inhibition markedly repressed the expression of xist and mir-7a-5p. The expression of xist was significantly up-regulated in myocardial cells in the LPS group compared with the control group, and significantly down-regulated in the LPS+ lncRNA^-^ group compared with the LPS group (**Figure 3**). Xist expression significantly up-regulated by lentivirus transfection in the LPS+ lncRNA^+^ group. Besides, there was no significant difference in the expression of xist among the LPS+ miRNA^-^, LPS+ miRNA^+^, and LPS groups. This implies that the suppression or overexpression of mir-7a-5p did not affect the expression of xist. The expression of mir-7a-5p was remarkably down-regulated in myocardial cells in the LPS and LPS+ miRNA^-^ groups compared with the control group. The expression of mir-7a-5p was significantly up-regulated in the LPS+ miRNA^+^ group. However, there was no remarkable difference in the expression of mir-7a-5p among the LPS+ lncRNA^-^, LPS+ lncRNA^+^, and LPS groups. This suggests that the suppression of or overexpression of xist did not affect the expression of mir-7a-5p (**Figure 3**).

### The correlation between non-coding RNA and PGC-1α

**Figure 4:**
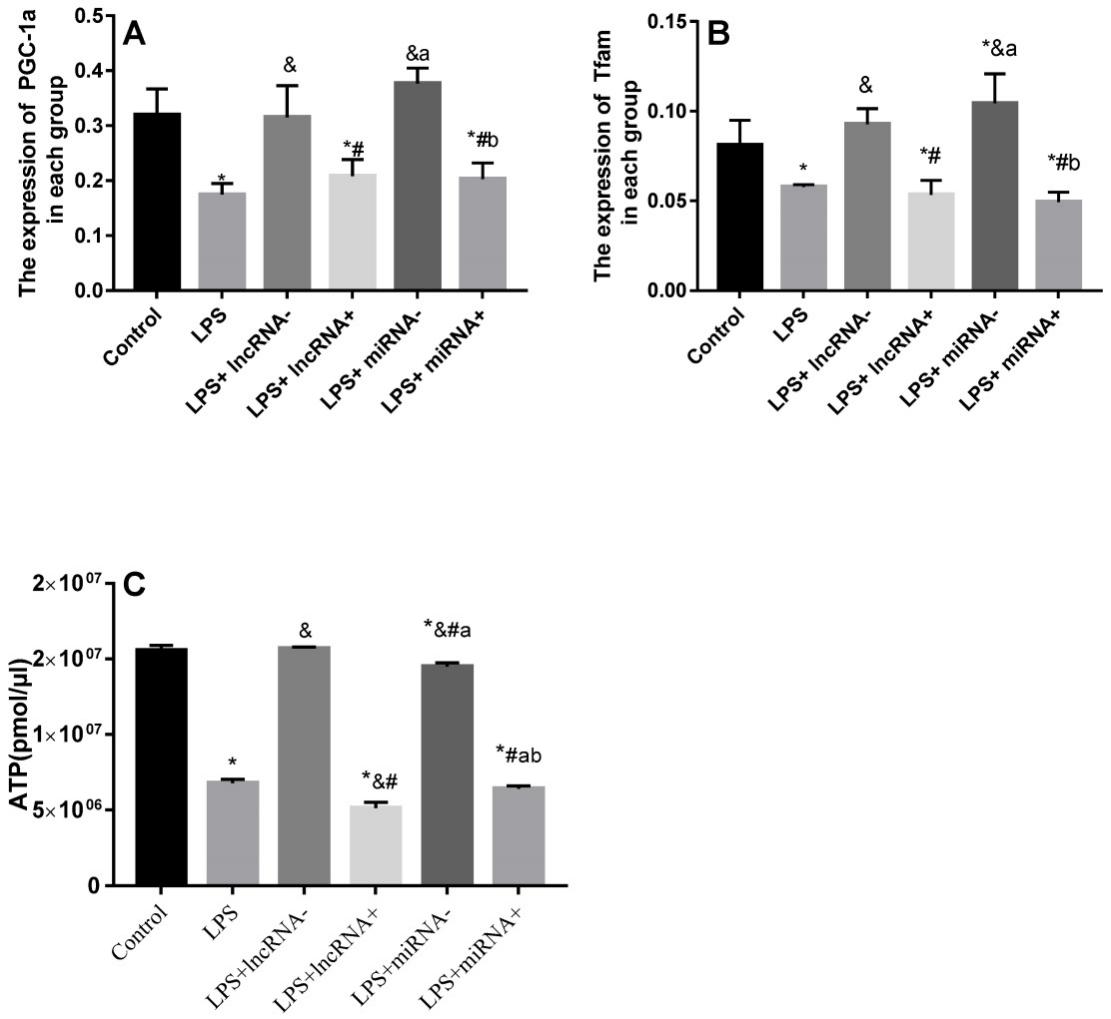
qRT-PCR analysis of RNA expression of the PGC-1α/Tfam signaling pathway. A: Expression levels of PGC-1α in each group. B: Expression levels of Tfam in each group. C: The total ATP content and expression levels of ATP in each group are shown. * *p* < 0.05 compared with the control group; & compared with the LPS group; # compared with the LPS+ lncRNA^-^ group; a compared with the LPS+ lncRNA^+^ group; b compared with the LPS+ miRNA^-^ group.

**Figure 5:**
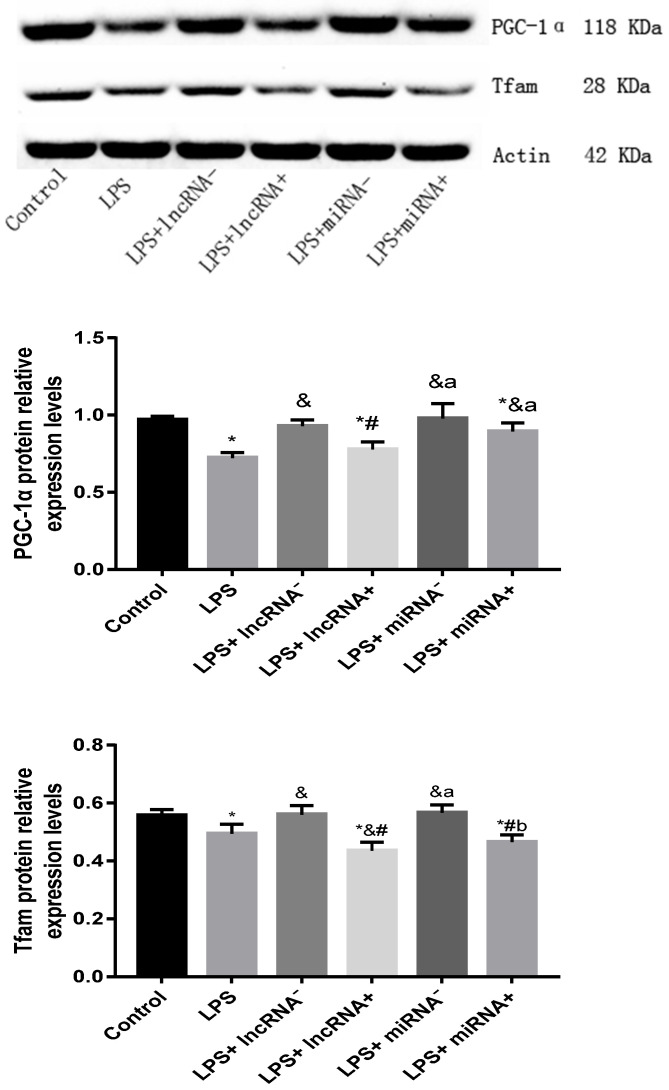
The protein expression of PGC-1α and Tfam assayed using western blot. * *p* < 0.05 vs. the control group; & *p* < 0.05 vs. the LPS group. # *p* < 0.05 vs. the LPS+ lncRNA^-^ group, a *p* < 0.05 vs. the LPS+ lncRNA^+^ group, b *p* < 0.05 vs. the LPS+ miRNA^-^ group.

Compared with the control group, the expression of PGC-1α was significantly down-regulated in the LPS, LPS+ lncRNA^+^, and LPS+ miRNA^+^ groups. The expression of PGC-1α was up modulated markedly after the suppression of xist and mir-7a-5p compared with the LPS group. Moreover, the repression of xist and mir-7a-5p was found to significantly up-regulate the expression of PGC-1α. The expression of Tfam was significantly down-regulated in the LPS, LPS+ lncRNA^+^, and LPS+ miRNA^+^ groups (*P <* 0.05), whereas this expression was up-regulated following the suppression of xist and mir-7a-5p. There was no remarkable difference in the expression levels of Tfam among the LPS+ lncRNA^+^, LPS+ miRNA^+^, and LPS groups. The suppression of xist and mir-7a-5p increased the expression levels of Tfam. Similar results were reported in Western Blotting analysis. (**Figure 4A-B & Figure 5**).

### Xist or mir-7a-5p impact cardiomyocyte ATP levels

The ATP levels are a vital representative of energy metabolism. Following LPS treatment, the levels of ATP were markedly reduced. Xist and mir-7a-5p inhibition resulted in a significant increase in ATP levels, but a remarkable reduction after xist and mir-7a-5p overexpression. The repression of xist and mir-7a-5p caused an increase in the levels of ATP (**Figure 4C**).

## Discussion

### Xist impact cardiomyocyte apoptosis

Xist plays an essential role in the proliferation, migration, apoptosis, and differentiation of various human cells 26. Findings from a recent study posit that xist in myocardial cells is up-regulated after myocardial infarction, and knockdown of xist protects against cell death induced by myocardial infarction 15. Moreover, xist is significantly up-regulated after spinal cord injury 27. PGC-1 α is a transcription coactivator, which interacts with many transcription factors involved in energy metabolism 28. Numerous types of miRNAs regulate mitochondrial function in various ways; for instance, Microrna-130b regulates mitochondrial biosynthesis via the PGC-1 α/Tfam axis 28. However, no studies have been conducted exploring the relationship between xist and the PGC-1 α pathway. In this study, increased xist expression was found to be associated with reduced PGC-1α and Tfam expression, and decreased ATP levels. This implies that repressing the expression of xist improves the expression of ATP, and provides insights for the treatment of sepsis. However, there are still some limitations to this study. Presently, the underlying mechanism between xist and PGC-1α remains unclear. The differences in ATP content may be driven by differences in cellular apoptosis. Therefore, further studies are needed to provide a better understanding of the relationship between xist and PGC-1 α pathway.

### Mir-7a-5p impact cardiomyocytes

Mir-7-5p plays different roles in different diseases. Overexpression of mir-7-5p remarkably represses cell proliferation and migration 29. In pancreatic ductal adenocarcinoma, mir-7-5p targets sox18 to suppress cell proliferation, migration, and invasion 30. Similarly, in melanoma cells, mir-7-5p inhibits cell proliferation and metastasis by repressing Rel A / NF - κB 31. In a myocardial ischemia-reperfusion model, the expression of mir-7-5p was significantly decreased 32,33. The repression of mir-7-5p improves injury induced by ANRIL depletion-exacerbated in hypoxic H9c2 cells 33. However, the mechanism of mir-7a-5p in sepsis cardiomyocytes remains unclear. In this study, increased expression of mir-7a-5p was found which was, consistent with reduced PGC-1α and Tfam expression, and reduced ATP levels. Therefore, the suppression of mir-7a-5p improves ATP expression. These changes indicated that xist and mir-7a-5p may modulate the expression of PGC-1α and Tfam in MCM cells. But we do not prove that the effects of xist and mir-7a-5p on murine cardiomyocyte apoptosis in response to LPS are PGC-1 alpha / Tfam dependent. This is clearly an important next step in this research.

### Reciprocal regulation between Xist and mir-7a-5p

The relationship between xist and mir-7a-5p was established using a database (http://starbase.sysu.edu.cn/index.php) (**Table 2**) and found that xist has a binding site of mir-7a-5p. However, this study showed that the expression of mir-7a-5p did not change after inhibition or overexpression of xist. Similarly, after inhibition or overexpression of mir-7a-5p, no significant difference was observed in the expression of xist, implying that there is no direct modulatory relationship between the two. Therefore, whether there are other regulatory mechanisms requires to be studied further. Overall, regulating xist alone did not affect the expression of mir-7a-5p; similarly, regulating mir-7a-5p alone did not affect the expression of xist.

In conclusion, the down-regulation of xist and mir-7a-5p improves myocardial damage. This study provides new insights for drug design for the treatment of sepsis induced-myocardial damage via modulation of the expressions of xist and mir-7a-5p. It should be noted, however, there are still differences between the experiment model of LPS-induced myocardial injury and the sepsis caused myocardial damage in clinical.

## Supplementary Material

Supplementary figures and tables.

## Figures and Tables

**Table 1:** Primer sequences

primer	Sequence
xist-F	TCACTCCTGCCTTTCGTGAC
xist-R	AACAAGTGGGGTGAGCACAA
Mir-7a-5p-F	CAGGCCACCTCTACAGGACA
Mir-7a-5p-R	AGGAACATGAGGAAGGTGTGAA
PGC-1α-F	TCAAGCCACTACAGACACCG
PGC-1α-R	TCGTGCTCTTTGCGGTATTC
Tfam-F	GGGAATGTGGAGCGTGCTAA
Tfam-R	TGATAGACGAGGGGATGCGA
ACTB-F	CATGTACGTTGCTATCCAGGC
ACTB-R	CTCCTTAATGTCACGCACGAT

**Table 2:** Underlined letters denote complementary sequences

	Sequence
Xist	5' ugcuuuAUAU-ACGAAGUCUUCCc 3'
mir-7a-5p	3' uguuguUUUAGUG-AUCAGAAGGu 5'

## Abbreviations

X-inactive specific transcript: Xist
Quantitative real-time PCR: qRT-PCR
Long noncoding RNA: lncRNA
Peroxisome proliferator-activated receptor-γ coactivator-1α: PGC-1α
Transcription Factor A, Mitochondrial: Tfam
mitochondrial DNA: mt-DNA
adenosine triphosphate: ATP
MicroRNAs: miRNAs
Delta-Opioid Receptor: DOR
Recombinant Human Interleukin-5: IL-5
interleukin-1B: IL-1B
lipopolysaccharides: LPS
standard deviation: SD
