# Supplementary material for: Down-regulation of Xist and Mir-7a-5p improves LPS-induced myocardial injury
Source: Int J Med Sci. 2020 Sep 16;17(16):2570–7. doi: 10.7150/ijms.45408 (PMC7532474; doi:10.7150/ijms.45408)
Supplement: Supplementary file 1 — Supplementary figures and tables. [file ijmsv17p2570s1.pdf]

Supplementary material-1

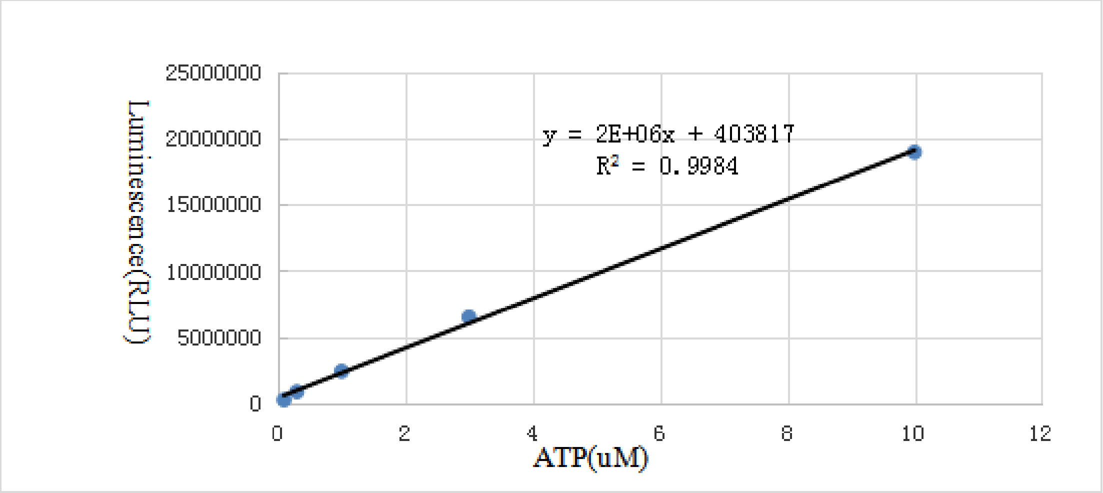

Figure 1: A series of ATP standards was measured to provide a calibration curve.

Supplementary material-2

Establishment of the sepsis cell model

We evaluated the morphology of the myocardial cells cultured under an ordinary optical microscope (100×). In the LPS group, the myocardial cells had margin changes(Figure1). Cell apoptosis was detected by flow cytometry (Figure2). This implies that the sepsis cell model was established successfully in the myocardial cells .

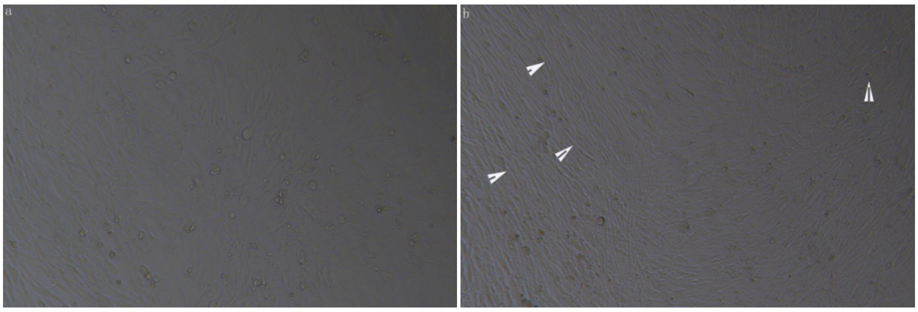

Figure.1 Observation of myocardial cell under an ordinary optical microscopy（100×）: a：Control group, b: LPS group; The cells margin changed in the LPS group.

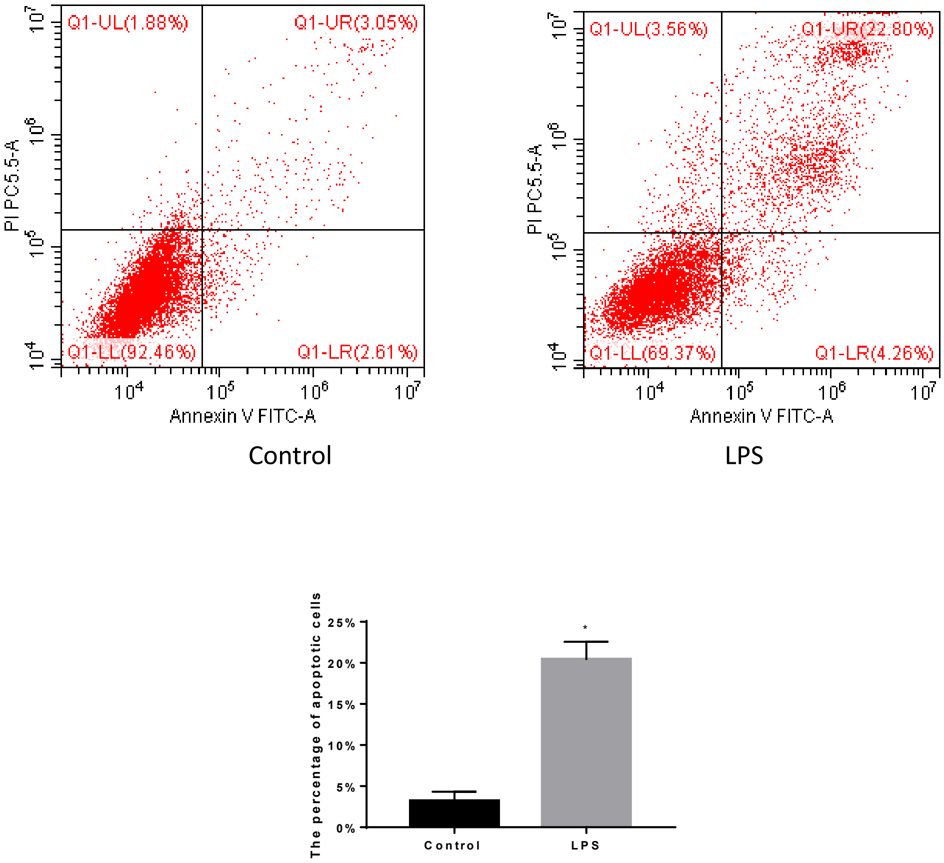

Figure.2 Increased cardiomyocyte apoptosis in MCM cells after the treatment with LPS. *$p < 0.05$ compared with the control group;
